# Dynamics of Plasma and Urinary Extracellular DNA in Acute Kidney Injury

**DOI:** 10.3390/ijms23063402

**Published:** 2022-03-21

**Authors:** Alexander Jančuška, Alena Potočárová, Alexandra Gaál Kovalčíková, Ľudmila Podracká, Janka Bábíčková, Peter Celec, Ľubomíra Tóthová

**Affiliations:** 1 Institute of Molecular Biomedicine, Faculty of Medicine, Comenius University, 81108 Bratislava, Slovakia; alexander.jancuska@gmail.com (A.J.); potocar.alena@gmail.com (A.P.); alexandra.kovalcikova114@gmail.com (A.G.K.); jana.babickova@gmail.com (J.B.); petercelec@gmail.com (P.C.); 2 Department of Paediatrics, National Institute of Children’s Diseases and Faculty of Medicine, Comenius University in Bratislava, 83340 Bratislava, Slovakia; podracka12@yahoo.com; 3 Department of Clinical Medicine, University of Bergen, 5021 Bergen, Norway; 4 Institute of Pathophysiology, Faculty of Medicine, Comenius University, 81108 Bratislava, Slovakia

**Keywords:** glycerol-induced nephropathy, non-invasive marker, cell-free DNA, mitochondrial DNA, rhabdomyolysis

## Abstract

Early and reliable markers of acute kidney injury (AKI) are essential. One such candidate marker of tissue damage is extracellular DNA (ecDNA). The aim of our present study is to describe the unknown dynamics of ecDNA in an animal model of AKI. Glycerol-induced nephropathy was used to model AKI in adult male Wistar rats (n = 93). Blood and urine samples were collected 1, 3, and 24 h after model induction. Total ecDNA and its sub-cellular origin was assessed. In the plasma, total ecDNA and nuclear ecDNA were significantly increased in the AKI group already after 1 h (160% and 270%, respectively, *p* = 0.02 and *p* = 0.04). Both nuclear and mitochondrial ecDNA were higher after 3 h (180% and 170%, respectively, *p* = 0.002 and *p* = 0.005). Urinary ecDNA concentrations in the AKI group were significantly increased only 24 h after model induction (130% for total ecDNA, *p* = 0.009; 210% for nuclear ecDNA, *p* = 0.02; and 200% for mitochondrial ecDNA, *p* = 0.0009). Our results indicate that plasma ecDNA has the potential to serve as an early and sensitive, albeit non-specific marker of AKI. Further studies should elucidate the source of ecDNA and the dynamics of ecDNA in other animal models of AKI and patients with AKI.

## 1. Introduction

Acute kidney injury (AKI) is a clinical syndrome characterized by the rapid decrease in kidney function, retention of nitrogenous waste products, acid-base and electrolyte balance abnormalities and other homeostasis impairments. Currently, it is defined as either a rise in serum creatinine (SCr) of ≥26.5 μmol/L (0.3 mg/dL) within 48 h, a 50% increase in SCr within 7 days or a urine output of <0.5 mL/kg/h for >6 h [1,2]. In addition, AKI is one of the main risk factors for the development of chronic kidney disease, linked to serious health and economic burden, including life-span shortening mainly through increased cardiovascular mortality [2,3,4].

In current clinical practice, the diagnosis of AKI is based on the rise in serum creatinine concentration. However, there is a time gap between the causative kidney insult and this rise, which means the loss of a potential therapeutic window. Additionally, the assessment of the rise in the serum creatinine may be troublesome as its concentration can be influenced by non-renal factors. Additionally, histologic studies have shown that kidneys can undergo detectable injury without inflicting any rise in serum creatinine concentration. About 50% of the glomerular filtration rate must be lost to exert a rise in the serum creatinine [5,6]. Thus, there is a need for better AKI biomarkers capable of the earlier detection or prediction of kidney damage before the deterioration of its function occurs [7].

Extracellular DNA (ecDNA) was first discovered in human plasma even before the structure of DNA was known. Since then, its role in the pathophysiology and as a marker in various diseases was found [8]. Moreover, ecDNA concentration and the size of its fragments differ in individuals with various diseases [9]. Increased ecDNA was described in several animal studies of AKI, e.g., in plasma in ischemia reperfusion injury [10] and in urine in sepsis-induced AKI [11]. In clinical practice, ecDNA in plasma was higher in septic patients with AKI compared to septic patients without it [12]. Similarly, ecDNA was a promising predictor of post-cardiac surgery AKI [13,14] and various post-kidney transplant complications [15]. The possibility of analyzing ecDNA in the urine is especially promising, since it is less invasive and more convenient for the patient than plasma sampling [16].

We have previously shown that plasma ecDNA is increased in AKI models of glycerol-induced nephropathy and bilateral ureteral obstruction (48 h period), and bilateral nephrectomy (24 h period) [17]. However, 24 and 48 h are quite long intervals, during which kidney damage might lead to the deterioration of its function. Thus, the time dynamics of ecDNA within 24 h after causing kidney insult should be better studied. Glycerol-induced rhabdomyolysis seems to be reliable model to induce AKI with subsequent increase in DNA without need of surgery [17,18]. Therefore, in the present study, we aim to elucidate the time dynamics of ecDNA during glycerol-induced nephropathy and investigate its potential as an early biomarker of AKI.

## 2. Results

### 2.1. Concentrations of Creatinine and Urea in the Plasma

**Figure 1:**
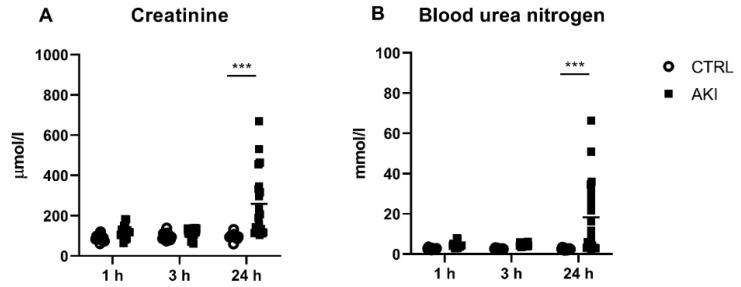
Concentrations of plasma creatinine (**A**) and urea (**B**) at different time points after AKI induction. *** denotes *p* < 0.001. CTRL—control group; AKI—glycerol-induced acute kidney injury.

Neither plasma creatinine nor BUN were significantly higher in AKI vs. CTRL groups 1 h and 3 h (early) after injury induction (Figure 1). Both creatinine (*p* = 0.0001; Figure 1A) and urea (*p* = 0.001; Figure 1B) were significantly different between the CTRL and AKI animals 24 h after AKI induction. The plasma creatinine concentrations were approximately 2.5-times higher (96 ± 5 µmol/L in CTRL vs. 260 ± 37 µmol/L in AKI group, *p* = 0.0001), while the plasma urea concentration was approximately 7-times higher in AKI than in the corresponding CTRL group 24 h after induction (2.6 ± 0.1 mmol/L in CTRL vs. 18.3 ± 4.1 mmol/L in AKI group, *p* = 0.0001).

### 2.2. Concentrations of ecDNA in the Plasma

**Figure 2:**
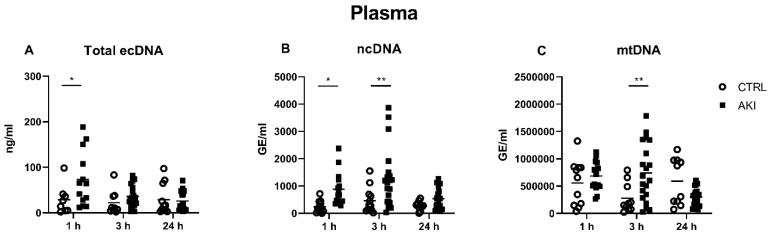
Concentrations of plasma total ecDNA (**A**), ncDNA (**B**) and mtDNA (**C**) at different time points after AKI induction. CTRL—control group; AKI—glycerol-induced acute kidney injury. * denotes *p* < 0.05. ** denotes *p* < 0.01.

One hour after the AKI induction, the total concentration of ecDNA in the plasma was significantly higher in the AKI group compared to the CTRL group (29 ± 11 ng/mL in CTRL vs. 73 ± 17 ng/mL in AKI group, *p* = 0.02). No significant differences between the groups were observed at the 3 h and 24 h time points (Figure 2A).

The concentration of plasma ncDNA was already 4-times higher, 1 h after AKI induction (237 ± 68 GE/mL in CTRL vs. 881 ± 156 GE/mL in AKI group, *p* = 0.04, Figure 2B), reaching the highest concentrations 3 h after AKI induction (459 ± 136 GE/mL in CTRL vs. 1276 ± 242 GE/mL in AKI group, *p* = 0.002).

The plasma concentrations of mtDNA showed different dynamics (Figure 2C). The highest increase was detected 3 h after the injury induction (277,104 ± 85,557 GE/mL in CTRL vs. 739,654 ± 117,902 GE/mL in AKI group, *p* = 0.005). At the 1 h time point, the increase was not significant (556,473 ± 125,468 GE/mL in CTRL vs. 685,212 ± 68,461 GE/mL in AKI group). There were no differences in mtDNA between the AKI and CTRL groups at the 24 h time point.

### 2.3. Concentrations of ecDNA in the Urine

**Figure 3:**
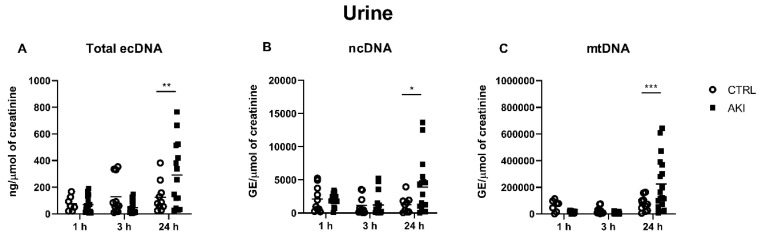
Concentrations of urinary total ecDNA (**A**), ncDNA (**B**) and mtDNA (**C**) per micromole of urinary creatinine at different time points after AKI induction. * denotes *p* < 0.05. ** denotes *p* < 0.01. *** denotes *p* < 0.001. CTRL—control group; AKI—glycerol-induced acute kidney injury.

In the urine samples, the total ecDNA concentration was significantly higher after 24 h, when compared to the control group (125 ± 36 ng/µmol of creatinine in CTRL vs. 292 ± 63 ng/µmol of creatinine in the AKI group, *p* = 0.009). The differences at other time points were not significant (Figure 3A).

The results show a significant difference in the ncDNA concentrations in the 24 h time point (1255 ± 417 GE/µmol of creatinine in CTRL vs. 3898 ± 991 GE/µmol of creatinine in the AKI group, *p* = 0.02). At the 1- and 3 h time points, the differences between the groups were not significant (Figure 3B).

In line with the results found in the plasma, mtDNA reached higher concentrations than ncDNA in the urine. Concentrations of mtDNA in the CTRL did not differ from those in the AKI group, 1 h and 3 h after the model induction. At 24 h after the induction, the concentration was significantly higher in the AKI group (74,643 ± 17,507 GE/µmol of creatinine in CTRL vs. 224,942 ± 47,018 GE/µmol in AKI group, *p* = 0.0009, Figure 3C).

### 2.4. Correlations between the AKI Functional Markers and DNA Measurements

Spearman’s correlation analyses revealed a negative correlation between plasma creatinine and ncDNA as well as mtDNA (ncDNA: r = −0.36, *p* = 0.009; mtDNA: r = −0.27, *p* = 0.04), while no correlation with total ecDNA was found (r = −0.02, *p* = 0.92). No significant correlation between BUN and plasma ecDNA was found (ecDNA: r = 0.006, *p* = 0.97; ncDNA: r = −0.23, *p* = 0.11; mtDNA: r = −0.17, *p* = 0.22). Additionally, we analyzed the potential association of plasma creatinine and urinary ecDNA. While the total ecDNA and ncDNA concentrations in the urine showed no significant association with plasma creatinine (ecDNA: r = 0.18, *p* = 0.20; ncDNA: r = 0.25, *p* = 0.09), urinary mtDNA correlated positively with plasma creatinine (r = 0.42, *p* = 0.002, Supplementary Table S1).

## 3. Discussion

In our animal experiment, we examined the rise in plasma and urinary ecDNA concentrations following glycerol nephropathy, with the time dynamics of total ecDNA, ncDNA and mtDNA. Additionally, plasma concentrations of nitrogenous waste products (creatinine and urea) were assessed as traditional markers of deteriorating kidney function, to confirm the functionality of our model, and to determine the markers that are superior for early diagnosis. Creatinine and urea concentrations significantly rose 24 h after the glycerol injection, but not at the early time points 1 h and 3 h after the injury. On the contrary, the mtDNA was higher 1 h and ncDNA 3 h after the model induction, which is much earlier than the creatinine or urea.

In AKI, ecDNA can originate from necrotic or apoptotic kidney tissue cells that are damaged by the primary kidney insult and extracellular traps (ETs) of activated immune cells that cause further tissue damage (and ecDNA release) and the promotion of immune activation, leading to the production of even more ETs [7,19,20]. In particular, experimental studies proved that neutrophil ETs are important in the mechanism of AKI caused by ischemia-reperfusion injury [19,20] while macrophage ETs take part in the pathogenesis of AKI resulting from rhabdomyolysis [18]. Our study analyzed the sub-cellular origin of ecDNA and its dynamics within a 24 h period, rather than tissue origin. Although the kidney filters small ecDNA fragments into the urine, this amount is not significant for the overall ecDNA clearance from the circulation [7]. Therefore, the rise in its concentration is rather caused by the heightened release resulting from tissue injury and the subsequent immune cell activation in either the kidney or the collapsed skeletal muscle. If we take into account the estimated ecDNA half-life (4–30 min to 1–2 h) [2,6,7], we should assume that the concentration of ecDNA originating from the muscle should rapidly decline, so the 3 h peak of ncDNA should be attributed to the kidney, rather than muscle, while the peak of mtDNA at the 1 h time point might truly originate from the muscle. Unfortunately, no studies comparing ncDNA and mtDNA as markers of skeletal muscle lysis were published, to the best of our best knowledge; however, a study evaluating the overall ecDNA as a marker of skeletal muscle lysis in sled dogs showed that ecDNA did not immediately rise in the dogs suffering from this condition [21]. Furthermore, in a murine model of glycerol nephropathy, histologically and serologically evidenced skeletal muscle lysis did not induce a rise in total plasma ecDNA 1 h after the glycerol injection, while there was a significant rise at the 24 h time point, which led the authors to conclude that the latter rise originated from the kidney [18].

As for the urinary ecDNA, the only significant rises showed 24 h post-injection, which was consistent in all the evaluated ecDNA subgroups. Considering that the plasma concentrations of ecDNA in the AKI group were not higher at this time point, the rise does not seem attributable to trans-renal DNA, but rather to a release from the kidney tissue or urinary tract. However, the absence of significant rises in either total ecDNA, ncDNA or mtDNA concentrations in the urine at the 1 h and 3 h time points challenge our previous conclusion that the rises in plasma ecDNA at these time points originated from the kidney. Our work provides a new insight into the dynamics of urinary mtDNA in AKI, which was expected to be a promising AKI biomarker, according to previous clinical as well experimental works, such as those by Whitaker et al., which used a model of ischemia-reperfusion, and Hu et al., using sepsis [17]. In these works, urine was collected within the first 18 h and within the period of 6–24 h after the AKI-inducing insult, respectively. In our experiment, however, the concentrations in the AKI group did not differ between the groups at the 1 h and 3 h time points, and only showed a significant rise at the 24 h time point. However, it is difficult to compare the results, as the experiments used different models of AKI.

Apart from animal experiments, diagnostic potential of ecDNA is also analyzed in clinical studies. Concentrations of ecDNA in patients undergoing pump-on cardiac surgery was significantly higher immediately after surgery in those patients who developed postsurgical AKI, in comparison to those without AKI. This increase was also detectable 3 days after surgery [14]. Higher levels of ecDNA in the peripheral blood was described in hemolytic uremic syndrome [22]. Urinary mtDNA correlated with the markers of tubular injury (kidney injury molecule 1-KIM-1, neutrophil gelatinase-associated lipocalin—NGAL) and with the severity of renal mitochondrial damage in patients with sepsis [11]. Similarly, after receiving kidney transplantation, urinary mtDNA correlated positively with NGAL and negatively with the glomerular filtration rate levels in patients. High ecDNA concentrations were found in patients with delayed allograft functions or with acute rejection [23,24]. Thus, ecDNA was shown to be potentially clinically usable as an early, non-invasive marker of kidney damage. Technical limitations related to the processing of urine samples, standardization of isolation protocols and normalization of the measured concentrations likely make it difficult to study urinary ecDNA. However, our results suggest that urinary ecDNA increases much later than in plasma. Thus, at least in the used model of AKI, the technical issues limiting reproducibility are not of major relevance.

The main limitation of our experiment is the use of glycerol nephropathy alone and no other model of AKI, which leaves unanswered questions regarding the true source of ecDNA, as well as the possible different dynamics of ecDNA resulting from other kinds of kidney injury. Other models of AKI, especially those that do not primarily harm organs other than the kidney, should be utilized, such as ischemia-reperfusion injury or bilateral ureteral obstruction. Nevertheless, surgical models per se are associated with muscle damage, since they require an abdominal or retroperitoneal incision that might be associated with increased ecDNA. Moreover, anesthesia or kidney decapsulation during a sham operation might influence the levels of ecDNA [25,26,27]. Thus, it seems that using chemically induced models is a better approach. Lastly, the histological confirmation of kidney injury was not performed in the present study.

In conclusion, based on our results, the elevation of total ecDNA, ncDNA and mtDNA in plasma precedes an increase in conventional markers, such as creatinine and urea. Thus, ecDNA serves as a potential early marker for the detection of AKI. However, further studies should better characterize ecDNA as a kidney function biomarker. The application of different doses of glycerol might describe the relation between plasma and urinary ecDNA, and the degree of kidney damage. Moreover, it should be elucidated whether the same pattern of a rise in ecDNA is present in other animal models of AKI, and especially in patients with AKI.

## 4. Methods

### 4.1. Study Design

This study was approved by the Ethics Committee of the Institute of Pathophysiology, Faculty of Medicine, Comenius University in Bratislava, Slovakia (Ethics Committee approval number 03/2020/SKU11016 and State Veterinary and Food Office number 860-3/2020-220), and was carried out in line with relevant national legislation. The study was carried out in compliance with the Animal Research: Reporting of In Vivo Experiments (ARRIVE) guidelines. Adult male Wistar rats (n = 93, Velaz Prague, Czech Republic) were used. The rats were kept in a controlled 12 h light/dark cycle room, with ad libitum food and water access, with constant temperature (22 ± 2 °C) and humidity (45–65%).

Animals were randomly divided into two groups. Rhabdomyolysis-induced acute kidney injury (AKI) was developed using glycerol (glycerol nephropathy). Treated rats (AKI group, n = 58) received an intramuscular injection of glycerol solution (50% *v*/*v* in physiological saline, 8 mL/kg, Sigma Aldrich, St. Louis, MO, USA), split into 2 doses in both hind limbs. Age- and sex-matched rats in the control group (CTRL, n = 35) received saline in the same manner.

One hour prior to sacrifice, the rats were placed in metabolic cages for urine collection. The animals were sacrificed at 3 different time points—1 h (n = 28; 11 CTRL and 17 AKI group), 3 h (n = 33; 12 CTRL and 21 AKI group) and 24 h (n = 32; 12 CTRL and 20 AKI group)—after the glycerol injection. Animals were not used repeatedly at different time points. Prior to sacrifice, blood was collected from the anaesthetized animals (ketamine 100 mg/kg + xylazine 10 mg/kg, i.p. using 20G cannula (B. Braun, Melsungen, Germany)). The blood samples were centrifuged at 1600× *g* for 10 min, and the plasma was stored at −20 °C until further analysis.

### 4.2. Biochemical Analysis

Urea and creatinine in the plasma were measured using commercial kits (Urea Nitrogen Colorimetric Detection Kit, Arbor Assays, Ann Arbor, MI, USA. and Creatinine Serum Low Sample Volume, Arbor Assays, Ann Arbor, MI, USA, respectively). Standard protocols recommended by the manufacturer were used.

### 4.3. DNA Isolation and Quantification

Blood samples were centrifuged at 1600× *g* for 10 min at 4 °C and the supernatant was further centrifuged at 16,000× *g* for 10 min at 4 °C in order to obtain supernatant free from apoptotic bodies and cell debris. This protocol was performed according to a study published by Dennis Lo et al. [28]. The urine samples were processed in the same way as plasma samples.

EcDNA isolation from the plasma and urine samples was performed using a QIAamp DNA Mini Kit, according to the manufacturer’s protocol (QIAamp^®^ DNA Mini and Blood Mini Handbook, Qiagen, Hilden, Germany). DNA was eluted into pure water. All samples were frozen at −20 °C until further analysis.

Total ecDNA was quantified fluorometrically using a AccuClear Ultra High Sensitivity dsDNA Quantitation kit (Biotium, Fremont, CA, USA) and the multimode reader Synergy H1 (Biotek, Winooski, VT, USA). To characterize the subcellular origin of ecDNA, real-time PCR targeting a nuclear (glyceraldehyde-3-phosphate dehydrogenase) and a mitochondrial (cytochrome b) gene was used with the following primers: GAPDH—gaaatcccctggagctctgt and ctggcaccagatgaaatgtg, and CYTB—cctcccattcattatcgccgcccttgc and gtctgggtctcctagtaggtctgggaa. A commercial mastermix for an SYBR green-based quantification of DNA (SsoAdvanced Universal SYBR Green Supermix, Bio-Rad, Hercules, CA, USA) was used with the following thermal cycling protocol (activation at 95 °C for 15 min, 40 cycles of denaturation at 94 °C for 15 s, annealing at 52 °C for CYTB and at 61 °C for GAPDH for 30 s, extension at 60 °C for 30 s) on the Mastercycler^®^ ep realplex Real-Time PCR System (Eppendorf, Hamburg, Germany). The specificity of the PCR was checked with melting curve analysis and electrophoresis of the products. PCR efficiency was between 90% and 110%; inter- and intra-assay variability were below 5%. Plasma and urinary concentrations of nuclear DNA (ncDNA) and mitochondrial DNA (mtDNA) were calculated using a calibration curve prepared by the dilution of DNA isolated from rat liver and expressed as genome equivalents (GEs) per ml of plasma or per µmol of creatinine in the urine.

### 4.4. Statistical Analysis

Data were analyzed using GraphPad Prism 8.0.2 (San Diego, CA, USA). To compare the concentrations of the measured markers between the CTRL and AKI groups, two-way ANOVA was used (one factor was the time point; the second factor was the treatment) with subsequent Sidak’s post hoc multiple comparisons. *p*-values less than 0.05 were considered as a limit level of significance. Data are presented as the means.

## Data Availability

The datasets used and/or analyzed during the current study are available from the corresponding author upon reasonable request.

## Supplementary Materials

The following supporting information can be downloaded at: https://www.mdpi.com/article/10.3390/ijms23063402/s1.
