# Supplementary material for: Dynamics of Plasma and Urinary Extracellular DNA in Acute Kidney Injury
Source: Int J Mol Sci. 2022 Mar 21;23(6):3402. doi: 10.3390/ijms23063402 (PMC8949705; doi:10.3390/ijms23063402)
Supplement: Supplementary file 1 [file ijms-23-03402-s001.zip › ijms-1614772-supplementary.pdf]

Supplementary Table S1: Relation between kidney functions markers and concentrations of ecDNA in plasma and urine.

**Variable**	Plasma ecDNA	Plasma ncDNA	Plasma mtDNA	Urinary ecDNA	Urinary ncDNA	Urinary mtDNA
Plasma creatinine	r=-0.02 p=0.91	**r=-0.36 p=0.009**	**r=-0.27 p=0.05**	r=0.18 p=0.20	r=0.25 p=0.09	**r=0.42 p=0.002**
BUN	r=0.06 p=0.97	r=-0.23 p=0.10	r=-0.17 p=0.22	r=0.17 p=0.25	r=0.27 p=0.06	r=0.22 p=0.12
